# Antidepressant Prescription to Children/Adolescents and Its Effects on the Cardiovascular System, Comprising the Actual Questions of Periodicity of the Checkups, Cooperation among Pediatricians, Family Doctors, Cardiologists and Children-Adolescent Psychiatrists

**DOI:** 10.15388/Amed.2025.32.1.15

**Published:** 2025-02-18

**Authors:** Reda Montvilaitė

**Affiliations:** Faculty of Medicine, Vilnius University, Vilnius, Lithuania

**Keywords:** Antidepressants, cardiovascular, side effects, cardiovascular risk, children and adolescents, selective serotonin reuptake inhibitors (SSRIs), serotonin-norepinephrine reuptake inhibitors (SNRIs), tricyclic antidepressants, psychotropic medication, antidepressants in pediatrics, atypical antidepressants, benzodiazepines, lipid metabolism, antidepresantai, širdies ir kraujagyslių sistema, šalutinis poveikis, širdies ir kraujagyslių sistemos rizika, vaikai ir paaugliai, selektyvūs serotonino reabsorbcijos inhibitoriai (SSRI), serotonino ir norepinefrino reabsorbcijos inhibitoriai (SNRI), tricikliniai antidepresantai, psichotropiniai vaistai, antidepresantai pediatrijoje, atipiniai antidepresantai, benzodiazepinai, lipidų metabolizmas

## Abstract

**Background:**

Pharmacological treatment is one of the most effective ways to help psychiatric patients with depressive disorders. However, prescription of antidepressants to children and adolescents creates controversial thoughts due to possible negative effects on the cardiovascular system. Despite being beneficial in controlling serious illnesses, there is less research done on the side effects of antidepressants which would require periodical checkups and cooperation among medical specialists. This literature review was completed to evaluate effects of antidepressants on the cardiovascular system and the necessity of regular assessment while treating children and adolescents.

**Aim:**

To review the cardiovascular effects of antidepressants prescribed to children and adolescents; to discuss the need for regular patient checkups with a multidisciplinary team: pediatricians, family doctors, cardiologists and children-adolescent psychiatrists.

**Methodology:**

Literature sources were selected from the *Pubmed, Google Scholar, Clinical Key*, and *Research Gate* databases by following dates from 2013 to 2024 while using the following keywords and their combinations: antidepressant, cardiovascular, side effects, cardiovascular risk, children and adolescents, selective serotonin reuptake inhibitors (SSRIs), serotonin-norepinephrine reuptake inhibitors (SNRIs), tricyclic antidepressants (TCAs), psychotropic medication, antidepressants in pediatrics, atypical antidepressants, benzodiazepines, lipid metabolism.

**Results:**

Antidepressants, including SSRIs, SNRIs, TCAs, atypical antidepressants and benzodiazepines, are associated with significant cardiovascular risks in children and adolescents. SSRIs, like citalopram and escitalopram, can disturb the heart rhythm by prolonging the QT interval, or increasing the risk of serious arrhythmias. SNRIs have been linked to an elevated blood pressure and heart rate. TCAs are known for their proarrhythmic effects, particularly in overdose situations, posing a high risk of sudden cardiac events. Atypical antidepressants like bupropion can cause cardiovascular disturbances, especially when overdosed. Additionally, less commonly prescribed benzodiazepines contribute to cardiovascular risks when combined with SSRIs during pregnancy, due to increasing the likelihood of congenital heart defects. These risks underscore the importance of careful monitoring, dosage management and thorough cardiovascular assessment when prescribing these medications to children, adolescents, and pregnant women. A team consisting of professional specialists – children-adolescent psychiatrists, cardiologists, pediatricians and family doctors – should detect long-term effects of pharmacotherapy by checking up the young patients regularly.

**Conclusions:**

The use of antidepressants in children and adolescents, though crucial for managing severe psychiatric disorders, raises significant cardiovascular safety concerns. SSRIs, SNRIs, TCAs, atypical antidepressants, and benzodiazepines have varying cardiovascular risks, especially in vulnerable youth populations and during the prenatal period. Given these risks, careful prescribing, close monitoring, creating guidelines and collaboration among healthcare providers are essential to ensure safe and effective treatment. Additionally, more research is needed to fully understand the long-term cardiovascular impacts of these medications in the pediatric population.

## Abstract (it)

**Įvadas:**

Farmakologinis gydymas yra vienas iš veiksmingiausių būdų padėti depresija sergantiems psichiatrijos pacientams. Tačiau antidepresantų skyrimas vaikams ir paaugliams kelia prieštaringų minčių dėl galimo neigiamo poveikio širdies ir kraujagyslių sistemai. Nepaisant to, kad antidepresantai yra nau-dingi kontroliuojant sunkias ligas, yra atlikta mažiau tyrimų apie šalutinį antidepresantų poveikį, dėl kurio reikia periodiškai tikrintis ir bendradarbiauti medicinos specialistams. Ši literatūros apžvalga atlikta siekiant įvertinti antidepresantų poveikį širdies ir kraujagyslių sistemai bei reguliaraus vertinimo būtinybę gydant vaikus ir paauglius.

**Tikslas:**

Apžvelgti vaikams ir paaugliams skiriamų antidepresantų poveikį širdies ir kraujagyslių sistemai; aptarti reguliaraus pacientų tikrinimo būtinybę bendradarbiaujant su daugiadalykine komanda: pediatrais, šeimos gydytojais, kardiologais bei vaikų ir paauglių psichiatrais.

**Metodika:**

Literatūros šaltiniai buvo atrinkti iš *Pubmed, Google Scholar, Clinical Key, Research Gate* duomenų bazės po 2013–2024 m. datų, vartojant raktinius žodžius ir jų derinius: antidepresantai, širdies ir kraujagyslių sistema, šalutinis poveikis, kardiovaskulinė rizika, vaikai ir paaugliai, selektyvūs serotonino reabsorbcijos inhibitoriai (SSRI), serotonino ir norepinefrino reabsorbcijos inhibitoriai (SNRI), tricikliniai antidepresantai (TCA), psichotropiniai vaistai, antidepresantai pediatrijoje, atipiniai antidepresantai, benzodiazepinai, lipidų metabolizmas.

**Rezultatai:**

Antidepresantai, įskaitant SSRI, SNRI, TCA, atipinius antidepresantus ir benzodiazepinus, vaikams ir paaugliams yra susiję su didele kardiovaskuline rizika. SSRI, tokie kaip citalopramas ir escitalopramas, gali sutrikdyti širdies ritmą, pailgindami QT intervalą, todėl padidėja sunkių aritmijų rizika. SNRI yra susiję su padidėjusiu kraujospūdžiu ir širdies susitraukimų dažniu. TCA žinomi dėl savo proaritminio poveikio, ypač perdozavus, keliančio didelę staigių širdies įvykių riziką. Atipiniai antidepresantai, pavyzdžiui, bupropionas, gali sukelti širdies ir kraujagyslių sistemos sutrikimų, ypač perdozavus. Be to, rečiau skiriami benzodiazepinai prisideda prie širdies ir kraujagyslių rizikos, kai nėštumo metu vartojami kartu su SSRI, padidindami įgimtų širdies ydų tikimybę. Ši rizika pabrėžia, kad, skiriant šiuos vaistus vaikams, paaugliams ir nėščioms moterims, svarbu atidžiai stebėti, valdyti dozę ir nuodugniai įvertinti širdies ir kraujagyslių sistemos būklę. Komanda, sudaryta iš profesionalių specialistų: vaikų ir paauglių psichiatrų, kardiologų, pediatrų ir šeimos gydytojų, turėtų nustatyti ilgalaikį farmakoterapijos poveikį reguliariai tikrindama mažuosius pacientus.

**Išvados:**

Antidepresantų vartojimas vaikams ir paaugliams, nors ir labai svarbus gydant sunkius psichikos sutrikimus, kelia didelį susirūpinimą dėl širdies ir kraujagyslių saugumo. SSRI, SNRI, TCA, atipiniai antidepresantai ir benzodiazepinai kelia skirtingą širdies ir kraujagyslių sistemos riziką, ypač pažeidžiamoms jaunimo grupėms ir prenataliniu laikotarpiu. Atsižvelgiant į šią riziką, siekiant užtikrinti saugų ir veiksmingą gydymą, labai svarbu tinkamai skirti vaistus, dėmesingai stebėti jais gydomus pacientus, kurti gydymo gaires ir bendradarbiauti sveikatos priežiūros paslaugų teikėjams. Be to, reikia atlikti daugiau tyrimų, kad būtų galima visapusiškai suprasti ilgalaikį šių vaistų poveikį vaikų ir paauglių širdies ir kraujagyslių sistemai.

## Introduction

Depressive disorders remain a common confirmed diagnosis for children and adolescents during recent years. As a major depressive disorder being the leading contributor to the burden of disease in young people aged 10–24 years, the main heterogeneous symptoms are often accompanied with impaired social and educational skills. On top of experiencing irritability, depressed mood and suppressed energy, anhedonia or worthlessness, children become at a higher risk of psychotropic substance misuse, obesity and suicide [1, 2]. Therefore, prescription of antidepressants with recommendations to attend psychotherapy sessions are considered with great importance and are the most efficient treatment option for the illness.

However, with pharmacological intervention still being the base to control pediatric depressive disorders, comes an inconclusive clinical tracking of the patient’s health safety. Although long-term effects of medications that are used for the treatment of adults are described broadly, there is less research done in exploring the side effects for pediatric patients.

One of the most reported negative effects for adults caused by antidepressants are cardiovascular system *impairments* [3]. Even though a longstanding connection between both treated and untreated psychiatric conditions in youth and future cardiovascular disorders has been *broadly observed* [4], the provided treatment influences the heart and blood vessels simultaneously. Occurrence of serious complications, such as bradycardia, tachycardia, hypertension, hypotension, orthostatic hypotension, electrocardiogram (ECG) changes, electrolyte abnormalities, reduced cardiac conduction and output, arrythmias or sudden cardiac death, have been registered for people even with no previous cardiovascular problems. Abnormalities of the cardiovascular system are administrated more carefully by adult psychiatrists by following an improved selection of antidepressants in the routine clinical practice.

Fortunately, recent literature provides more information about changes in children’s cardiovascular system after the use of antidepressants. The latest insights suggest that pharmacological interventions may exert an impact on a child even before being born. In 2023, Ting Wu *et al*.’s study [5], the authors presented latest evidence on how prenatal drug exposure could be linked to cardiovascular system diseases in the postnatal period. Based on molecular mechanisms, the article highlighted that the use of maternal antidepressants 3 months before pregnancy as well as an early pregnancy exposure to the medication would elevate the risk of congenital heart disease among the offspring. For instance, such cardiovascular defects as defected heart septum formation was determined to newborns after exposure to some SSRIs.

Directly prescribed medication to depressive children and teenagers in the postnatal period also demonstrates a clinical significance of cardiovascular health. Consequences of the use of antidepressants may vary in relation to such factors as the specific type of medication, its dosage, the duration of treatment, and the individual characteristics of the patient. In this article, several classes of these medications (SSRIs, SNRIs, TCAs, Atypical antidepressants, and benzodiazepines) shall be presented individually, including not only those commonly prescribed, but also the options which are infrequently advised to choose for pediatric patients. Diverse cardiovascular features have been witnessed to appear after antidepressant usage, such as various electrocardiogram (ECG) changes, arrhythmias, hypertension or orthostatic hypotension and serotonin syndrome, and even indirect ones via lipid metabolism. Following the latest literature findings in antidepressants impacting the cardiovascular system, the important aspect of check-ups by doctors shall be reviewed.

## Purpose

The purpose of this article is to review the latest published literature and explore the data on the cardiovascular effects of antidepressants prescribed to children and adolescents and to discuss the need for regular patient checkups with a multidisciplinary team: pediatricians, family doctors, cardiologists, and children-adolescent psychiatrists.

## Study Material and Methods

This literature review was conducted by using the *Pubmed, Google Scholar, Clinical Key*, and *Research Gate* databases. Publications were selected from 2013 to 2024 by using the following keywords and their combinations: antidepressant, cardiovascular, side effects, cardiovascular risk, children and adolescents, selective serotonin reuptake inhibitors (SSRIs), serotonin-norepinephrine reuptake inhibitors (SNRIs), tricyclic antidepressants (TCAs), psychotropic medication, antidepressants in pediatrics, atypical antidepressants, benzodiazepines, lipid metabolism. Following the selection process, studies not containing topic-related information, studies published earlier than 2013, studies targeting different age groups, and those not reporting medication side effects were excluded. Initially, 33 investigations were included in total.

## Results

### Selective Serotonin Reuptake Inhibitors (SSRIs)

The most common class of antidepressants prescribed for children experiencing symptoms of either mild or severe depression is SSRI. First line medications, such as fluoxetine, sertraline and escitalopram [4, 6], are frequently used to treat the *Major Depressive Disorder* (MDD) and other intermittent psychiatric illnesses strongly associated with an increased risk of suicide, such as *Generalized Anxiety Disorder* (GAD), or *Post-Traumatic Stress Disorder* (PTSD). However, there are only a few SSRIs approved by the FDA for pediatric patients, and, considering the lack of sufficient testing in randomized controlled trials, there is little research oriented towards specific pediatric indications [7, 8] As a result, serious adverse effects, including cardiovascular features, may occur in pediatric population after exposure to antidepressants. Additionally, disorganized patients’ consumption of SSRIs, irregular check-ups and the confusion experienced by clinicians in a generally unsettled period of antidepressants pharmacotherapy raise concerns, too [8, 9].

Recent studies suggest that even a mother’s exposure to SSRIs can impact the fetus and predict severe cardiovascular problems in postpartum. SSRIs are linked to a slight increase in preterm birth, persistent pulmonary hypertension of the newborn (PPHN), developmental disorders, and neonatal intensive care unit admissions [5]. While SSRIs elevate the risk of PPHN, the magnitude of this risk varies between different SSRI types, with sertraline posing the lowest risk for PPHN, and potentially making it the safest SSRI option during pregnancy in this regard.

Moreover, maternal antidepressant consumption before and during pregnancy increases the risk of congenital heart disease in the offspring [5]. Although the risk was considered negligible, intrauterine fluoxetine exposure was associated with a slightly elevated risk of several congenital malformations. However, a more pronounced risk was observed for cardiovascular defects, especially septal defects, among newborns that were prenatally exposed to fluoxetine. Furthermore, genetic factors, such as cytochrome P450 polymorphisms, can also influence the risk of cardiac birth defects by affecting how the mother metabolizes drugs. Despite the research linking antidepressants to birth defects, long-term adult cardiovascular outcomes remain understudied. However, some of the latest research consistently associates this treatment with the altered aortic function and reduced nitric oxide levels. The discovery confirmed that prenatal fluoxetine consumption may result in reducing aortic contractions in the offspring’s aorta. Therefore, the necessity to reevaluate prescription to mothers diagnosed with depression and to consider the mitigation of the side effects of SSRIs by using such compounds as *Suberoylanilide Hydroxamic Acid* (SAHA) is important in order to to restore the mitochondrial function and prevent their offspring from experiencing cardiovascular risks.

Furthermore, pediatric patients might experience certain *Electrocardiogram* (ECG) changes caused by SSRIs. In 2013, Angela S. Czaja *et al*. conducted a comparative study, where pediatric SSRIs users were compared in terms of the antidepressants they were prescribed so that to determine different risk potential for ventricular arrhythmias, cardiac arrests or sudden death. The results presented more frequent incidents of adverse cardiac outcomes to citalopram and escitalopram users rather than to fluoxetine users [10]. Another study conducted by Julie M. Zito *et al*. (2017) noted sudden motor vehicle death cases of adolescents and also found an association between the increased need for cardiac-related emergency visits and young users of antidepressants [11]. However, while assessing other cohorts, they noticed no relations between serious cardiovascular events and current stimulant exposure. Nevertheless, later studies confirmed that adolescents who were prescribed such SSRIs as Citalopram or Escitalopram could experience rhythmical disruptions, especially QT interval prolongation which can predispose fatal reentrant tachycardias known as Torsades de Pointes [12]. The least QT interval changes were noticed after exposure to Sertraline, whereas Paroxetine and Escitalopram caused high QTc values [13]. Therefore, due to being acknowledged as dose-dependent drugs, it is emphatically important to prescribe these SSRIs carefully and to contemplate the doses typically recommended for children.

SSRIs, the primary antidepressant class for children, could pose significant cardiovascular risks. Prenatal SSRI exposure is linked to a range of adverse outcomes, from preterm birth and cardiac defects to long-term cardiovascular problems. Furthermore, SSRIs can induce heart rhythm disturbances in some individuals, especially adolescents. These findings underscore the need for careful evaluation and monitoring when prescribing SSRIs not only to children but to pregnant women as well.

### Serotonin-Norepinephrine Reuptake Inhibitors (SNRIs)

Whereas SSRIs are the first line medication, serotonin-norepinephrine reuptake inhibitors (SNRIs), such as venlafaxine and duloxetine, may be considered as an alternative treatment for depressive and anxiety disorders. However, more side effects are being reported in comparison with SSRIs [14].

Research suggests that both SSRIs and SNRIs demonstrate positive results in adolescents, and neither of them is recommended for pre-pubertal children [15]. There is limited information comparing SSRIs to SNRIs assiduously, but SNRIs indicated slightly greater cardiovascular adverse effect rates than SSRIs. The collected body of research explored Venlafaxine, which resulted in clinically important changes for some patients. A significant elevation in the supine pulse rate, diastolic and systolic blood pressure, sometimes even lasting for three consecutive visits at least seven days apart, was mentioned [14]. Due to Venlafaxine expressing meaningful changes in the total blood pressure and the heart rate during short-term treatment, they might not be recommended as a long-term treatment choice.

Overall, SNRIs are not recommended as first line treatment according to the available data that present more frequent cardiovascular events [15]. Although the clinical significance of these effects in children and adolescents is still not defined at scientifically reliable levels, SNRIs have been potentially associated with hypertension and tachycardia. Thus, further research and comparative analysis with similar pharmacokinetic antidepressants are needed to elucidate the nature and magnitude of these risks. Following the usage of Venlafaxine in short-term anxiety treatment, and considering SNRIs’ compelling influence on the cardiovascular system, careful SNRI prescription and regular medical monitoring are recommended, especially during the initiation or adjustment of an antidepressant therapy, or when appended cardiovascular factors are manifested.

### Tricyclic Antidepressants (TCAs)

TCAs, such as amitriptyline and imipramine, are less commonly prescribed for pediatric patients today due to their side effect profile [1, 14]. TCAs are known to exert anticholinergic effects which can manifest as tachycardia, orthostatic hypotension, and conduction abnormalities in an electrocardiogram (ECG).

In Cardy *et al*.’s (2017) article, several studies were referenced in which children with depression or anxiety disorders were given either Imipramine, Paroxetine, or placebo treatment [1]. Unfortunately, research was withdrawn due to adverse cardiac events which the majority of the participants experienced, and provoked tachycardia or arrhythmia were reported. As a result, TCAs held no significant benefit over placebo for treating depression as well as prescribing desipramine to ADHD pediatric population. On the contrary, Amitriptyline was as effective as Gabapentin for treating pediatric neuropathic pain and improving sleep with no difference in negative events reported.

Since TCAs tend to have more severe side effects than the new generation antidepressants, they pose an elevated risk of intoxication. TCAs may have proarrhythmic effects, particularly in overdose situations, leading to arrhythmias and sudden cardiac death. In 2023, Anar Gurbanov *et al*.’s article discussed urgent cardiovascular disruptions caused by TCAs and found cardiac toxicity (supraventricular and ventricular arrhythmias) to be most common [16]. Whilst comparing old and new generation antidepressants, TCAs remain one of the leading drugs resulting in poisoning when overdosed, yet they comprise an important part of intensive care. Therefore, the key role in preventing severe cardiovascular events is weighing the risks of toxicity at therapeutic doses and overdose if depressive disorders are resilient to the usual SSRIs treatment [17]. Nevertheless, SSRIs demonstrate an increased risk of suicide-related behaviors, but fatal outcomes with SSRIs overdose are significantly rarer than TCAs overdose [18, 19].

However, when other antidepressants do not provide relief from severe depression, TCAs may help. Although they expose a narrow therapeutic index along with potential for serious cardiac toxicity, TCAs are generally reserved for rare cases where other antidepressant options have been ineffective or are contraindicated.

### Atypical Antidepressants

Atypical antidepressants, such as Bupropion and Mirtazapine, exhibit unique pharmacological profiles distinct from SSRIs, TCAs, and SNRIs. Therefore, these drugs are considered as an option when the patient suffers not only from depression but has an eating disorder, sleep problems, or suffers from a behavior disorder. Despite the more trustworthy impact on adults, neither Bupropion nor Mirtazapine have undergone rigorous testing in children yet, and, consequently, these agents should be considered only once other first-line treatments have failed [7**]**.

Based on older clinical trials which researched Bupropion’s cardiovascular effects, Hartford *et al*. conducted a simulation trial for the pediatric emergency staff in 2019 [20]. Bupropion overdose has been reported to cause several cardiovascular disturbances in pediatric population, such as tachycardia, QRS prolongation, and QT prolongation in children under 6 years old. Nevertheless, although the unintentionally ingested drug can possibly cause serious cardiac conditions in younger children, the occurrence rate is very low, and it minimally affects the cardiac conduction. Although, the pediatric emergency crew should be aware of a potential Bupropion overdose in children due to its extended-release pharmacokinetics.

In a case described by Tanidir *et al*. (2015), Mirtazapine was explored for possible adverse effects in treating adolescents with arrythmia and cardiomyopathy [21]. A 16-year-old patient developed depression, anxiety and significant weight loss, and, regarding the primary genetic syndrome that caused cardiac disabilities, Mirtazapine was chosen. The treatment was successful, and no adverse cardiac effects appeared.

On the contrary, another pilot study published in 2017 by Uchida *et al*. examined cardiovascular safety of nontricyclic antidepressant medications (non-TCAs) [22]. Both bupropion and mirtazapine as well as SSRIs and SNRIs were included in the trial. It was noted that psychiatrically referred participants aged from 6 to 17 years did not experience any significant adverse events. Weight- and age-corrected doses of the chosen medications did not alter the ECG and blood pressure parameters. Even though the sample size was small, the authors found no association between atypical antidepressants and negative cardiac effects in the pediatric population.

While atypical antidepressants offer potential benefits in treating depression with comorbid conditions, their use in children remains limited. Limited research and potential for adverse cardiac effects, particularly with Bupropion overdose, necessitate caution. The current evidence is mixed, and more rigorous trials are essential, but Bupropion and Mirtazapine could be considered when first-line antidepressant options have been exhausted. Careful monitoring and awareness of the potential drug interactions and individual risk factors are crucial when prescribing these medications to young patients.

### Benzodiazepines

This class of antidepressants is rarely prescribed to children, but some articles disagree with the current practice guidelines which turn down benzodiazepines when managing acute anxiety [23, 24]. Because of the increased abusive consumption in youth, they are safe as short-term anxiolytics to relieve preprocedural stress and are used to treat some neurological disorders.

Although standing out as addictive after prolonged consumption, Soto-Insuga *et al*.’s 2022 article analyzed the recommended doses and their adverse features for patients experiencing prolonged seizures [25]. For instance, 80% of occurrences are treated with first-line medication – benzodiazepines – and therefore there is a higher risk of arterial hypotension. Another study by Elmowafi *et al*. (2024) reported a minimal chance of a cardiac event (not exceeding 2%) among the researched children [26]. Female adolescents (aged 15–19 years) who were exposed to anxiolytics (especially Diazepam) mostly experienced fainting or collapse due to arterial hypotension.

Moreover, the prenatal exposure to benzodiazepines and their potential effect on the offspring’s cardiovascular system was pointed out in Wu *et al*.’s research in 2023 [5]. While single use of this medication did not appear to be associated with congenital malformations, a combination of benzodiazepines and SSRIs leads to a higher risk of disrupted heart development. Notably, certain genetic variants in the mother, such as Cytochrome 450 polymorphism, have been proven to be involved in regulating maternal drug side effects on embryonic heart development. Therefore, the combined antidepressants raise caution in drug administration during pregnancy even though no evidence compares the relative risk of benzodiazepines with SSRIs alone. So, the increased risk of SSRIs or their collaboration with benzodiazepines continue to be unclear due to a lack of comparative studies.

Pediatric psychiatrists rarely prescribe benzodiazepines. Even though they are highly effective for short-term treatment of severe stress, their addictive nature necessitates caution. While adolescents possibly can experience arterial hypotension, prenatal exposure to a combination of benzodiazepines and SSRIs raises awareness. While a single use likely poses no risk, together with SSRIs, the risk of congenital heart defects in the baby may increase. Further research is needed to understand the relative risks of benzodiazepines alone, and how a mother’s genetics might influence these risks.

### Lipid Metabolism

Some antidepressants may affect lipid metabolism, although the clinical significance of these effects in children and adolescents is less understood [27]. In 2024, Liu *et al*. presented a study of youth population with major depressive disorders where patients aged between 8 and 18 were analyzed [28]. One of the factors influencing the lipid levels to change was the therapeutic doses of antidepressants, taking fluoxetine as a coefficient. Increased levels of cholesterol and triglycerides have been reported in children using this medication. As a result, antidepressants proved to cause a higher risk of dyslipidemia and weight increase in youth which could potentially contribute to cardiovascular risk over the long term, especially when antidepressants are combined with antipsychotics [29].

Another study conducted by Bozdag *et al*. in 2024 explored most frequently prescribed SSRIs – Sertraline and Citalopram – and how they affect lipid metabolism through adipogenesis [30]. Although the research was conducted *in vitro* by using human mesenchymal cells, the findings suggested that SSRIs cause upregulation in adipocytes. Excessive synthesis of phospholipids and lysosomes leads to implications in maintaining a healthy metabolic balance. Consequently, the latest data on cytologic level could reveal more information of how psychopharmacotherapy affects the cardiovascular system through lipid metabolism pathways.

### Prescription and Check-ups

Prescription of pharmacological treatment for youth has been slowly decreasing in modern pediatric psychiatry. In recent years, research has revealed biased studies, financial conflicts of interest, and questionable research practice. As a result, there have been many concerns towards the safety and effectiveness antidepressants for children [31]. Despite the increased awareness, antidepressants among adolescents and young adults are thus, generally, significantly over-consumed. Furthermore, the overuse of antidepressants and their off-label prescribing for conditions not approved by regulators have not been the only issue.

However, while the availability for ‘proper’ psychotherapy that is accompanied by psychotropic treatment remains unreachable for the people most in need, doctors influenced by economic and social factors still usually choose to prescribe medication.

Children in foster care, juvenile justice systems, residential treatment facilities and those with intellectual or developmental disabilities tend to be treated in high-risk regime and often off-label. Therefore, organized and successful treatment of these patients is hardly ever achieved at all. Stutzman *et al*. in a 2021-dated article discussed these issues following the importance of landmark trials, pediatric specific guidelines and state-driven initiatives that could improve the development of psychopharmacology in the pediatric field and reduce the rates of over-prescription [32]. In addition, the authors raised attention towards the possible risk of negative effects that could increase in the long term. The lack of data about distant neurobiological, physical and social changes during a child’s development in combination with the already known short-term effects (growth velocity, reduced bone marrow, weight gain and risk for type 2 diabetes mellitus) lead to deprescribing the antidepressants.

Regarding the limited amount of evidence for the efficacy and possible serious side effects of antidepressants in the youth population, monitoring these patients is undoubtedly needed. However, the duration and frequency of regular check-ups after prescribing the medication are still not being discussed globally among specialists. In 2020, *Jack et al*. delivered a study in which the authors presented the data of UK children patients visiting pediatrists or psychiatrists before and after the first treatment prescription [33]. Less than half of the research participants were consulted by general practitioners (GPs) who can initiate antidepressant treatment, and the majority got their medications prescribed without the relevant specialist involved at all. While the first group visited their doctor less than 12 months before or 6 months after their first antidepressant initiation, others could not access the required mental health services because of the long waiting times and other difficulties. Nevertheless, almost half of the patients potentially were not prescribed the antidepressants by evidence-based indication. Therefore, further research is required to investigate GPs’ choice to prescribe psychotropic medication to children without a strict check-up regime and the overall practice barriers to adherence to the clinical guidelines.

The use of antidepressants in children and adolescents is a complex issue with growing concerns. Despite the research raising doubts about the safety and effectiveness of these medications, their use and off-label prescribing remain high, while affecting vulnerable youth groups. There is little knowledge of long-term negative effects, including research on the cardiovascular system. Although there is a lack of official evidence-based guidelines and agreements on how doctors should monitor newly prescribed patients, evidence suggests the importance of deprescribing practices to minimize potential harm for children. Moreover, the current follow-up appointments before and after considering psychopharmacotherapy is insufficient. Only robust methodologies and an improved access to qualified mental health professionals collaborating with pediatricians and children’s cardiologists would help in determining the efficacy and safety of antidepressants.

## Discussion and Study Limitations

The use of antidepressants in children and adolescents, while necessary for managing severe psychiatric disorders, raises significant concerns regarding the cardiovascular safety.

Recent literature describes such antidepressant classes as SSRIs, SNRIs, TCAs, atypical antidepressants and benzodiazepines that have been linked to various cardiovascular disruptions, ranging from rhythmic heart disturbances to long-term cardiovascular risks. SSRIs have been reported to cause various heart malformations prenatally when mothers are prescribed this class of medication [5]. Some reports link SSRIs to an altering heart rhythm in some adolescents [10]. Even though the amount of the so-far presented data is scarce, these findings underscore the need for careful evaluation and monitoring when prescribing not only SSRIs to the youth postnatally, but to pregnant women as well. While SSRIs are still perceived as being the first-line treatment for psychiatric conditions, SNRIs are recommended when the patient experiences SSRIs-resistant disorders or acute cardiovascular changes after exposure to SSRIs. Yet, SNRIs have a potential to cause disturbances in the blood flow and the heart rate, and therefore SNRIs require similar attention, especially given their generally higher side effect profile compared to SSRIs [14]. TCAs, due to their earlier highlighted pro-arrhythmic properties and a narrow therapeutic index, are generally reserved for cases where other treatments have already failed. Current research mostly reports TCAs-induced cardiovascular changes and cardiac attacks in pediatric emergency rooms; consequently, doctors and parents should observe children more attentively so that to prevent unintentional overdose or suicidal behavior [16]. Atypical antidepressants, though studied less in pediatric populations, show mixed results regarding the cardiovascular safety, emphasizing the need for further research [20]. Bupropion is easier overdosed, and there are articles that are contradictory regarding the issue if QRS or QR prolongation is significant towards children. Nevertheless, Bupropion and Mirtazapine offer potential benefits in treating depression with comorbid disorders, but they are only prescribed when the first-line treatment has already been exhausted. Further research with a larger sample size is needed to explore atypical antidepressants’ cardiovascular effects more deeply.

Benzodiazepines, though effective particularly for stress relief before procedures and *status epilepticus*, also demand careful consideration due to their addictive potential and a possible impact on the cardiovascular system when used in combination with SSRIs during pregnancy. Despite detecting congenital heart malformations appear after maternal consumption of SSRIs with benzodiazepines, follow-up research is still necessary to decide if benzodiazepines alone have relevant impact on a baby’s cardiovascular system. Additionally, antidepressants’ indirect ways of affecting the cardiovascular system of children are explored through lipid metabolism in a study dated 2024 [30]. The increased risk for dyslipidemia and weight gain even when prescribing the recommended pediatric doses were associated with higher chances for cardiovascular changes in the future.

Given these concerns, it is imperative that the prescription of antidepressants in children and adolescents should be accompanied by rigorous and regular cardiovascular monitoring, especially when prescribed to youth and pregnant women. When other mental health options are unavailable for economically and socially vulnerable children, psychopharmacology is still vastly considered as the main solution to psychiatric issues. Therefore, current literature supports the idea of deprescribing when the use of antidepressants is unnecessary. Collaboration among pediatricians, psychiatrists and cardiologists is essential to ensure the safe and effective use of these medications.

Although this review provides a comprehensive overview of how different antidepressants affect the cardiovascular system in the pediatric population, it is still subject to several limitations. Primarily, the existing published literature is very limited, and it evidently contains some methodological flaws that cause difficulties in evaluating the results. While the research with the adult population is broadly analyzed, the young population is studied less because of the treatment recommendations to avoid long-term prescription or refrain from certain types of antidepressants and rather promote psychotherapeutic treatment. Therefore, it creates a scarcity of information impacting any potential long-term pediatric specific research. Secondly, the studies were performed with limited sample sizes. Also, the selected literature mentions the challenges posed by off-label prescribing or lack of standardized monitoring that could influence the outcomes. Additionally, the recent discoveries on medications affecting the genetic level need to be expanded more so that to draw more definitive conclusions and to help establish more detailed recommendations for children health specialists.

To conclude, there is a pressing need for more complete research in order to fully understand the long-term cardiovascular impacts of antidepressants on pediatric patients. The development of evidence-based guidelines for their use is needed as well as regular monitoring of the newly introduced patients. Overall, the importance of continuous check-ups with the doctors before and after adequate prescription is noted. However, with the objective to minimize significant cardiovascular adverse effects, it is crucial to raise more discussions on the latest research.
